# Performance of the Patient Health Questionnaire-4 (PHQ-4) in four latin American countries: rethinking the dimensionality of the scale from the perspective of network psychometry

**DOI:** 10.1186/s41155-026-00399-w

**Published:** 2026-06-06

**Authors:** Lindsey W. Vilca, Aaron Travezaño-Cabrera, Claudio Rojas-Jara, Rodrigo Moreta-Herrera, Diana Ximena Puerta-Cortés, Martin Noe-Grijalva, Daniel E. Yupanqui-Lorenzo, Sergio Chacón-Armijo, Evelyn Cuesta-Andaluz

**Affiliations:** 1 Universidad Señor de Sipán, Chiclayo, Peru; 2 Escuela Profesional de Psicología, Facultad de Ciencias de la Salud, Universidad Peruana Unión, Lima, Peru; 3 Universidad Católica del Maule, Maule, Chile; 4 Pontificia Universidad Católica del Ecuador, Quito, Ecuador; 5 Universidad de Ibagué, Ibagué, Colombia; 6 Universidad César Vallejo, Trujillo, Peru; 7 Universidad Científica del Sur, Lima, Peru; 8 Universidad Católica de Temuco, Temuco, Chile; 9 Universidad Internacional De La Rioja, Logroño, Spain

**Keywords:** Anxiety, Depression, Network Psychometrics, Cross-cultural Invariance, Network stability, Latin America

## Abstract

**Background:**

Anxiety and depression frequently co-occur and represent a major global mental health burden, highlighting the need for efficient and psychometrically sound screening tools. The Patient Health Questionnaire-4 (PHQ-4) is an ultra-brief instrument widely used across cultures; however, evidence regarding its internal structure and cross-cultural equivalence from a psychometric network perspective remains limited, particularly in Latin America. The study aims to investigate the psychometric properties of the PHQ-4 from the perspective of psychometric networks in four Latin American countries.

**Methods:**

A total of 1,887 participants aged 18 to 40 years were included in the study, distributed across the following countries: Chile (*n* = 353), Colombia (*n* = 467), Ecuador (*n* = 373), and Peru (*n* = 499).

**Results:**

The EGA analysis showed that the items form a single community composed of four nodes with large network loadings in all four countries. The UVA analysis demonstrated that all items are relevant to the network model in all the countries analyzed. Evidence of structural consistency and item stability was also found, with the items being stable and systematically organized into a single community across all countries. Furthermore, the bootEGA analysis demonstrated that the PHQ-4 exhibits metric invariance across countries.

**Conclusions:**

The results of this study provide solid evidence of the psychometric performance of the PHQ-4 in the four countries studied. Therefore, a single-community structure is proposed to explain the internal structure of the PHQ-4.

## Introduction

Anxiety and depression are two of the most prevalent and disabling mental disorders worldwide, with a rising incidence affecting diverse populations. These conditions often occur together, demonstrating a marked association (Tunç et al., 2023). These disorders have a significant impact on quality of life, cognitive functioning, academic and occupational performance, interpersonal relationships, and physical health (World Health Organization, 2022). Their etiology is multifactorial, influenced by biopsychosocial factors such as chronic stress, socioecosystemic instability, and adverse childhood experiences (Kessler et al. 2005a, b; Peng et al. 2024; Thomson et al. 2021). Furthermore, their impact transcends the psychological realm, as they are primary risk factors for the development of cardiovascular disease, type 2 diabetes, and increased premature mortality (Bobo et al., 2022; Charlson et al., 2018). In its most severe form, depression is a key predictor of suicide, one of the leading causes of death among young people and adults (Liu, 2023; Nock et al., 2013).

In recent years, both the prevalence and severity of anxiety and depression symptoms have increased, generating a growing demand for mental health services. The health crisis caused by the COVID-19 pandemic exacerbated this situation, increasing the prevalence and severity of symptoms in diverse populations, highlighting the urgent need to strengthen mental health intervention strategies (Bueno-Notivol et al., 2021). Furthermore, the treatment time required by these patients has increased, limiting the capacity of health systems to treat new cases and contributing to the saturation of psychological services (American Psychological Association, 2023). In addition, the high comorbidity between anxiety and depression complicates both their diagnosis and treatment, increasing the burden on health professionals and society in general (Baxter et al., 2014; Vasiliadis et al., 2024). This scenario presents a significant challenge for healthcare systems, underscoring the pressing need for more effective and accessible interventions to address these disorders worldwide.

In this context, the early and accurate identification of anxiety and depression becomes crucial for implementing effective interventions. The use of ultra-brief screening instruments emerges as an efficient and practical solution, particularly in clinical and research settings where resources and time are limited (Lantos et al., 2023; Rentzsch et al., 2022). These instruments stand out for their rapid application, which favors greater individual participation and reduces fatigue during the assessment (Passarelli et al., 2024). Importantly, despite their brevity, they maintain adequate psychometric properties, achieving satisfactory levels of reliability and validity that compare favorably with longer versions (Hadžibajramović et al., 2024; Rentzsch et al., 2022).

Furthermore, by reducing the cognitive and emotional burden on participants, ultra-brief instruments not only increase response rates but also improve the quality of the data collected by reducing errors and dropout rates. However, their application is not without challenges, such as the potential loss of detailed information and lower precision in individual measurement, which could limit their applicability in contexts requiring a comprehensive assessment of symptoms (Monticone et al., 2021; Passarelli et al., 2024). Despite these limitations, ultra-brief instruments are presented as valuable tools for screening and monitoring anxiety and depression symptoms, facilitating timely interventions without significantly compromising the validity of the assessment.

In this context, unlike extensive scales designed to assess anxiety and depression in a detailed manner, such as the Beck Anxiety Inventory (BAI-II), the Beck Depression Inventory-II (BDI-II), the State-Trait Anxiety Inventory (STAI), or the Center for Epidemiologic Studies Depression Scale (CES-D), which exceed 20 items and provide an in-depth analysis of symptoms, there are shorter instruments that optimize administration time without significantly compromising psychometric validity. Examples of these are the Patient Health Questionnaire-9 (PHQ-9) and the Generalized Anxiety Disorder-7 (GAD-7), both of which have fewer than 10 items and are widely used in clinical and research settings, with extensive verification in South America (Moreno-Montero et al., 2025). However, when seeking an even faster and more efficient assessment of both conditions, the Patient Health Questionnaire-4 (PHQ-4) represents an ultra-brief alternative, with only four items, allowing for effective initial screening for anxiety and depression in various settings.

The PHQ-4 has proven to be a valid and reliable instrument in multiple studies that have evaluated its consistency in both clinical and non-clinical samples. International evidence supports the factorial validity of this model, with studies identifying one- and two-factor structures, the latter being the most commonly reported and with support for invariance across variables such as sex, age, and gender (Caro-Fuentes & Sanabria-Mazo, 2024). In Latin America, both models were evaluated in twelve countries, finding that the bifactorial model presented adequate fit indices. At the same time, the unidimensional model also showed a good fit, except in the RMSEA, where it presented a greater measurement error (Caycho-Rodríguez et al. 2024a, 2024b, 2024c). Although the two-factor proposal is consistent across various studies (Kazlauskas et al., 2023; Obeid et al., 2024), in the Latin American research, the correlation between factors was high (greater than 0.80 in most cases), which would suggest the presence of a common factor or a strong relationship between the indicators. Because of this, it has been deemed necessary to explore its functioning using other analytical methods that allow a more in-depth evaluation of its structure and precision. This is especially relevant given that the PHQ-4 is a robust instrument used to measure anxiety and depression, both as a predictor and dependent variable, which further supports its usefulness in the assessment of emotional symptoms. In response to the need to explore alternative psychometric methods, evidence on the use of the PHQ-4 within psychometric network models remains limited. A recent study conducted in Paraguay used exploratory graph analysis (EGA) to identify a single community (Caycho-Rodríguez et al. 2024a, 2024b, 2024c). Resampling techniques revealed 100% stability in the network structure associated with the unidimensional model, suggesting that the PHQ-4 is a psychometrically stable instrument. Although several studies have utilized this tool to investigate the relationship between anxiety and depression, as well as other variables (Caycho-Rodríguez et al. 2024a, 2024b, 2024c; Ramm et al. 2025; Yupanqui-Lorenzo et al. 2024), the study of its structure from the perspective of network psychometrics still requires further expansion.

There are advantages to analyzing instruments using psychometric networks because it allows for the visualization and interpretation of dynamic relationships between symptoms rather than assuming rigid latent structures (Borsboom, 2017). Unlike classical factor models, which assume that symptoms reflect an underlying construct, network analysis posits that symptoms can activate each other, providing a more nuanced understanding of the mechanisms underlying anxiety and depression (Robinaugh et al., 2020). Furthermore, assessing the centrality of symptoms within networks allows for the identification of the most influential elements in the symptom structure, which has direct implications for the development of more effective clinical interventions (Fried et al., 2017; Fried & Cramer, 2017). The application of network analysis to the study of the PHQ-4 would not only strengthen its structural validity but also contribute to a more precise understanding of symptomatic coactivation patterns in different populations, optimizing its usefulness in the early detection and clinical follow-up of anxiety and depression. Furthermore, assessing invariance in psychometric networks is crucial to ensure the stability of relationships between items across different groups, thereby avoiding biased interpretations. Without this analysis, comparisons may be flawed and compromise the validity of the findings, highlighting the importance of its inclusion in psychometric studies (Jamison et al., 2024; van Borkulo et al., 2023).

In that sense, the study objectives focus on (a) reporting the internal structure-based validity of the PHQ-4 using a psychometric network-based approach, (b) examining network stability as a key indicator to assess the internal consistency of the PHQ-4, providing additional evidence on its structural consistency and stability, and (c) evaluating the invariance of the PHQ-4’s network structure across four Latin American countries, which will determine whether the instrument maintains its consistent psychometric structure across different cultural and geographical contexts. These objectives aim to address a critical gap in existing literature, which highlights the scarcity of studies on the PHQ-4 from a psychometric network perspective.

## Method

### Participants

Participants were recruited from each country using a non-probability convenience sample, following the inclusion criteria: (a) adult, (b) nationality from one of the four countries studied, (c) use of technological means, and (d) signing informed consent. Exclusion criteria were: (a) failure to complete all tests and (b) leaving sociodemographic data blank.

Table 1 shows that the sample consisted of 1,887 participants whose ages ranged from 18 to 60 years. The average age in Peru was 26.04 years (*SD* = 8.75), in Chile it was 26.99 years (*SD* = 9.74), in Colombia it was 25.32 years (*SD* = 9.9), and in Ecuador it was 27.43 years (*SD* = 8.2). The majority of participants were women, representing 63.35% in Peru, 75% in Chile, 60.49% in Colombia, and 68.47% in Ecuador. Regarding marital status, the majority were single, with 85.64% in Peru, 77.97% in Chile, 76.22% in Colombia, and 74.88% in Ecuador. Regarding the level of education, the majority had higher education in Peru (76.06%), Chile (83.91%), Colombia (41.01%), and Ecuador (73.89%).

**Table 1:** Sociodemographic data of participants from Peru, Chile, Colombia and Ecuador

Variables	Peru	Chile	Colombia	Ecuador	Total
	*N* = 543	*N* = 404	*N* = 534	*N* = 406	*N* = 1887
Sex					
Male	199(36.65%)	101(25%)	211(39.51%)	128(31.53%)	639(33.86%)
Female	344(63.35%)	303(75%)	323(60.49%)	278(68.47%)	1248(66.14%)
Marital Status					
Single	465(85.64%)	315(77.97%)	407(76.22%)	304(74.88%)	1491(79.01%)
Married	42(7.73%)	39(9.65%)	63(11.8%)	72(17.73%)	216(11.45%)
Widowed	6(1.1%)	0(0%)	3(0.56%)	3(0.74%)	12(0.64%)
Divorced	4(0.74%)	8(1.98%)	16(3%)	10(2.46%)	38(2.01%)
Cohabiting	26(4.79%)	42(10.4%)	45(8.43%)	17(4.19%)	130(6.89%)
Educational Level					
No Education	1(0.18%)	0(0%)	0(0%)	0(0%)	1(0.05%)
Primary	0(0%)	1(0.25%)	1(0.19%)	2(0.49%)	4(0.21%)
Secondary	76(14%)	38(9.41%)	202(37.83%)	75(18.47%)	391(20.72%)
Technical	53(9.76%)	26(6.44%)	112(20.97%)	29(7.14%)	220(11.66%)
Higher	413(76.06%)	339(83.91%)	219(41.01%)	300(73.89%)	1271(67.36%)

## Measures

### Patient Health Questionnaire-4 (PHQ-4)

PHQ-4 is an ultra-brief instrument developed in the United States (Kroenke et al., 2009) to detect the presence of anxiety and depression symptoms. The questionnaire includes two items from the Patient Health Questionnaire (PHQ-2) and two items from the Generalized Anxiety Disorder Screener (GAD-2). The psychometric properties of the instrument have been extensively studied in several countries (Christodoulaki et al., 2022; Obeid et al., 2024; Wicke et al., 2022). In the present study, the adapted version was used in several Latin American countries, including Peru, Colombia, Chile, and Ecuador (Caycho-Rodríguez et al. 2024a, 2024b, 2024c).

The questionnaire consists of four response categories: Not at all (0), Several days (1), More than half the days (2), and Almost every day (3). The instrument does not present inverse items; therefore, a higher score on the scale would show a greater presence of anxiety and depression indicators.

### Procedure

Data collection was conducted between March 2024 and November 2024 in Peru, Chile, Colombia, and Ecuador using an online questionnaire administered through Google Forms. The survey link was distributed via social media platforms (e.g., Facebook, WhatsApp) and email invitations, following a non-probability convenience sampling approach. Participants accessed the questionnaire voluntarily and completed it anonymously. The average completion time was approximately 15 min.

The Google Forms consisted of three sections: The first section provided information about the study objectives and informed consent. The second section collected information on the participants’ sociodemographic data, and the third section presented the items from the PHQ-4 scale. Only participants who voluntarily agreed to participate in the first section were able to complete the other sections of the form.

Additionally, the researcher distributed online forms via social media and email in all countries. It is worth noting that a researcher was responsible for coordinating the collection in each country. Participants took approximately 15 min to complete the entire online form. This study adhered to the standards outlined in the Declaration of Helsinki (World Medical Association, 2013), and informed consent was obtained from all participants before they participated in the study.

### Data analysis

First, a descriptive analysis of the items was performed, reporting the item response rate and Spearman’s correlation matrix. Second, Unique Variable Analysis (UVA) was estimated to determine the presence of redundant items. For this purpose, Weighted Topological Overlap (wTO) was used, where values greater than 0.25 indicate that the items exhibit local dependence (redundant items), based on simulation-based cutoff recommendations for wTO with EBICglasso (Christensen et al. 2023a, b).

Third, Exploratory Graph Analysis (EGA) (Golino & Epskamp, 2017) was used to assess the internal structure of the instrument. For this process, A Spearman correlation matrix was used as input, as recommended for ordinal data in network psychometrics due to its robustness to non-normality and stable performance in GGM estimation (Isvoranu & Epskamp, 2023). The network model used was the Gaussian Graphical Model (GGM), and the EBICglasso algorithm (Epskamp & Fried, 2018) was used to estimate the GGM network structure, as it estimates a regularized GGM based on a correlation matrix as input, combining the LASSO (glasso) graphical algorithm (Friedman et al., 2008) with the Extended Bayesian Information Criterion (EBIC) (Chen & Chen, 2008) to fine-tune parameter selection. The number of communities present in the GGM network was determined using the Louvain algorithm, as it is one of the most accurate and unbiased methods in combination with the EBICglasso method (Christensen, Garrido, Guerra-Peña et al., 2023). Additionally, network loads were calculated, considering small (0.15), moderate (0.25), and large (0.35) network load values (Christensen and Golino 2021a).

Fourth, structural consistency and item stability were assessed using the Bootstrap Exploratory Graph Analysis (bootEGA) approach, where 1000 resamples were used to determine the structural consistency and stability of the items, with values greater than 0.75 being considered acceptable (Christensen and Golino 2021b). Fifth, measurement invariance was studied through a series of steps. Initially, configural invariance was estimated, for which the data were clustered into groups, and a common sample structure was identified using bootEGA and itemStability. If any variable has a replicate less than 0.70 in its assigned dimension, it is considered unstable and, therefore, not invariant. Therefore, configural invariance allows us to be certain that the same nodes cluster in the same communities for all groups (Jamison et al., 2024). Metric invariance was then estimated using the permutation test, which allows us to verify whether the network loadings are equal across groups. This procedure ensures that the network structure and its connections are measured consistently across groups. To determine the lack of metric invariance, it is recommended to assess the significance of item values using both the p-value (< 0.05) and the corrected p-value (p_BH<0.10) (Jamison et al., 2024).

Additionally, a complementary analysis of practical unidimensionality was conducted using the closeness to unidimensionality framework (Ferrando & Lorenzo-Seva, 2018). This analysis included the estimation of ECV-global, I-ECV, and IREAL indices. This complementary approach allows evaluating whether the PHQ-4 can be treated as essentially unidimensional in practical terms, without assuming strict unidimensionality or equivalence with latent variable models.

Statistical analyses were performed using R (R Core Team, 2019) and RStudio (RStudio Team, 2018). The following packages were used for network analysis: EGAnet (Golino & Christensen, 2024), qgraph (Epskamp et al., 2012), and bootnet (Epskamp et al., 2018).

## Results

### Descriptive analysis

Table 2 shows the descriptive statistics for the sample from Peru, Chile, Colombia, and Ecuador. Specifically, the correlation matrix indicates that all items exhibit moderate relationships, ranging from 0.48 to 0.56 in Peru. A similar pattern is observed in Chile (0.42 to 0.70), Colombia (0.51 to 0.62), and Ecuador (0.53 to 0.63). Likewise, all items showed a median of 1 and low IQR values, indicating a concentration of responses in the lower categories. Additionally, skewness and kurtosis values were within acceptable ranges (g1 < ± 2; g2 < ± 7), and the response distribution showed a higher concentration in categories 0 and 1.

**Table 2:** Descriptive analysis of the items

Peru	1	2	3	4	Md	IQR	g1	g2	RNL	Response rate			
										0	1	2	3
1	-				1	1	0.79	0.02	0.51	37.6%	42.9%	13.8%	5.7%
2	0.56	-			1	1	0.76	-0.29	0.52	42.4%	35.7%	16.6%	5.3%
3	0.48	0.50	-		1	1	0.77	-0.22	0.45	42.4%	37.0%	16.0%	4.6%
4	0.56	0.55	0.54	-	1	1	0.85	0.04	0.55	41.8%	39.4%	13.4%	5.3%

Chile	1	2	3	4	Md	IQR	g1	g2	RNL	Response rate			
										0	1	2	3
1	-				1	1	0.46	-0.51	0.55	16.8%	49.8%	20.3%	13.1%
2	0.70	-			1	2	0.59	-0.47	0.51	30.9%	42.1%	17.8%	9.2%
3	0.42	0.42	-		1	1	0.78	-0.17	0.43	35.4%	42.6%	13.1%	8.9%
4	0.53	0.49	0.65	-	1	1	0.84	-0.12	0.57	39.6%	39.4%	12.9%	8.2%

Colombia	1	2	3	4	Md	IQR	g1	g2	RNL	Response rate			
										0	1	2	3
1	-				1	1	0.81	0.11	0.54	35.4%	45.3%	12.7%	6.6%
2	0.62	-			1	1	0.87	0.14	0.50	40.6%	41.2%	12.5%	5.6%
3	0.55	0.51	-		1	1	0.86	0.23	0.46	37.6%	44.6%	11.8%	6.0%
4	0.60	0.60	0.62	-	1	1	0.87	-0.02	0.58	40.4%	39.5%	12.4%	7.7%

Ecuador	1	2	3	4	Md	IQR	g1	g2	RNL	Response rate			
										0	1	2	3
1	-				1	1	0.92	0.54	0.48	40.9%	44.8%	10.1%	4.2%
2	0.62	-			1	1	1.01	0.59	0.54	46.3%	40.1%	9.9%	3.7%
3	0.53	0.55	-		0	1	1.06	0.44	0.48	51.7%	33.7%	11.3%	3.2%
4	0.57	0.62	0.63	-	1	1	1.06	0.64	0.58	47.5%	38.7%	9.6%	4.2%

Total	1	2	3	4	Md	IQR	g1	g2	RNL	Response rate			
										0	1	2	3
1	-				1	1	0.74	-0.04	0.52	33.2%	45.5%	14.1%	7.2%
2	0.62	-			1	1	0.81	-0.06	0.52	40.3%	39.6%	14.3%	5.9%
3	0.50	0.50	-		1	1	0.86	0.06	0.46	41.5%	39.6%	13.2%	5.6%
4	0.56	0.56	0.61	-	1	1	0.91	0.12	0.56	42.2%	39.3%	12.2%	6.4%

0: Not at all,1: Several days, 2: More than half the days, 3: Nearly every day, Md: Median, IQR: Interquartile range; g1 = skewness; g2 = kurtosis; RNL: Revised Network Loadings

## Validity Based on Internal Structure

**Fig. 1:**
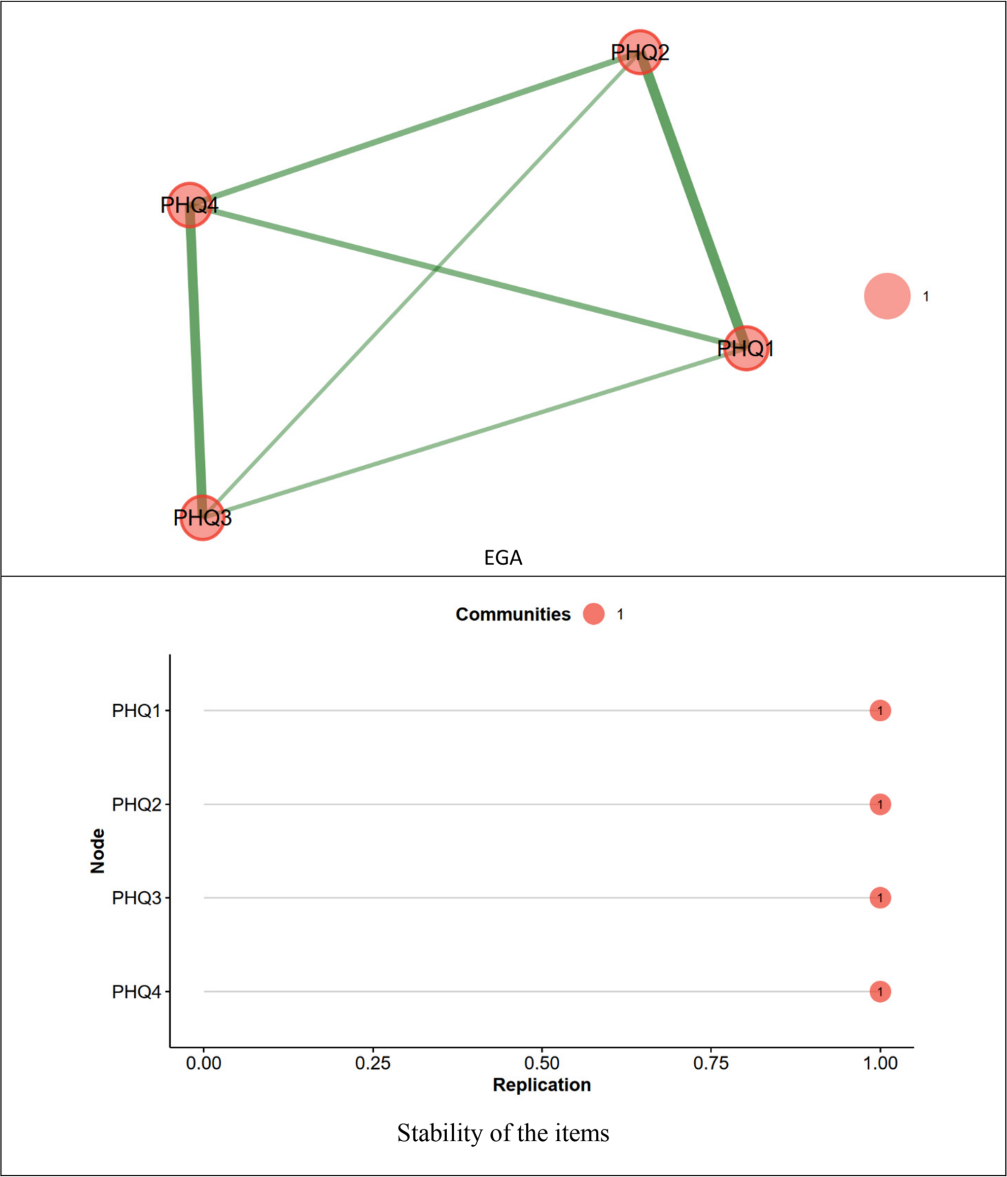
EGA and Stability of the items in the total sample

Figure 1 illustrates the dimensionality estimated using the EGA, which identified a community comprising four nodes. The interconnections between the nodes are outlined by green lines, demonstrating positive correlations, and the intensity of the networks is reflected in the thickness of the lines. In the total sample, In the total sample, the analyzed network consisted of four nodes and six edges, indicating a fully connected network (density = 1), where all possible edges are present. The strength of the associations between nodes varied, with edge weights ranging from 0.144 to 0.385.

**Fig. 2:**
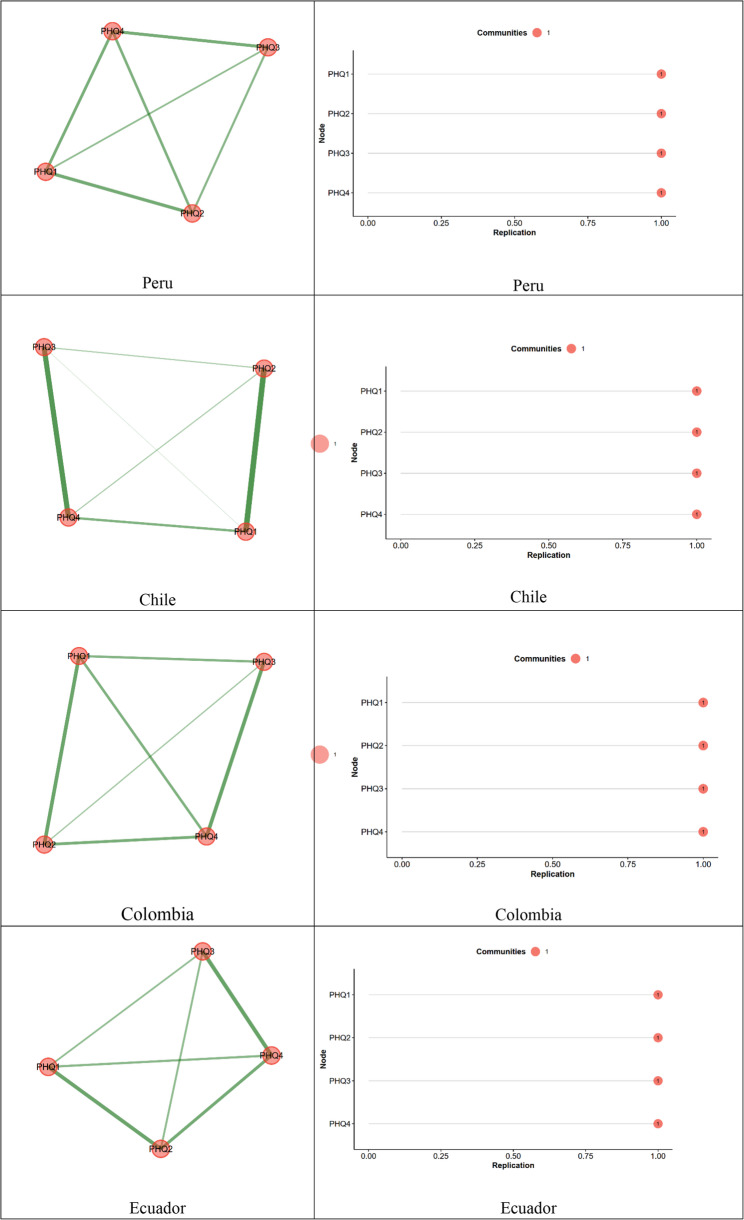
Exploratory graph analysis of the PHQ-4 by country

Figure 2 shows that the analyzed network in Peru has four nodes and six edges, indicating a fully connected network (density = 1), where all nodes are connected. The strength of the associations between nodes varied, with edge weights ranging from 0.171 to 0.294. Similarly, in Chile, the analyzed network was identified to have four nodes and six edges, demonstrating a density of 1, indicating a fully connected network, where all nodes are connected. The edge weights showed variability from 0.039 to 0.532. Regarding the Colombian model, the analyzed network was found to have four nodes and six edges, demonstrating a density of 1, indicating a fully connected network, where all nodes are connected. The strength of associations between nodes varied from 0.115 to 0.352. Regarding the Ecuadorian model, the analyzed network was found to have four nodes and six edges, indicating a density of 1, indicating a fully connected network, where all nodes are connected. The strength of associations between nodes varied from 0.169 to 0.359. Regarding the network load values reviewed, high values (> 0.35) were found in all countries (see Table 2).

As a complementary analysis, the closeness to unidimensionality indices were examined to assess the extent to which the PHQ-4 can be considered essentially unidimensional in practical terms. The closeness to unidimensionality analysis provided strong evidence supporting a dominant general dimension. The ECV-global value was 0.933, indicating that a large proportion of the common variance is explained by a single factor. At the item level, the I-ECV values ranged from 0.906 to 0.952, suggesting that all items contribute strongly to the general dimension. Regarding residual multidimensionality, the IREAL-global value was 0.218, and all IREAL-item values were below the 0.30 threshold, indicating that secondary factor influences are limited. Overall, these results support the interpretation that the PHQ-4 can be considered essentially unidimensional in practical terms.

### Structural consistency and item stability

Figure 1 shows that, in the total sample, item stability showed acceptable values (≥ 0.75), maintaining the initial structure of the EGA. Likewise, structural consistency revealed that the only community identified by EGA was replicated in 100% of cases. Furthermore, Fig. 2 shows that for Peru, Chile, Colombia, and Ecuador, adequate values were obtained for item stability (≥ 0.75) as well as for structural consistency, where the initial structure identified by EGA was replicated in 100% of cases. These results indicate high structural consistency and item stability, as the identified structure was replicated in 100% of the bootstrap samples.

### Measurement invariance

To assess measurement invariance across countries, configural invariance was first established, which enabled us to verify that the community and nodes were comparable across countries (see Fig. 2). Additionally, bootEGA was run on the entire sample, providing evidence of consistent clustering of nodes within a community (see Fig. 1). These results provided evidence of configural invariance, as the community remained stable across countries. Once this procedure was completed, the metric invariance analysis was performed across countries.

**Fig. 3:**
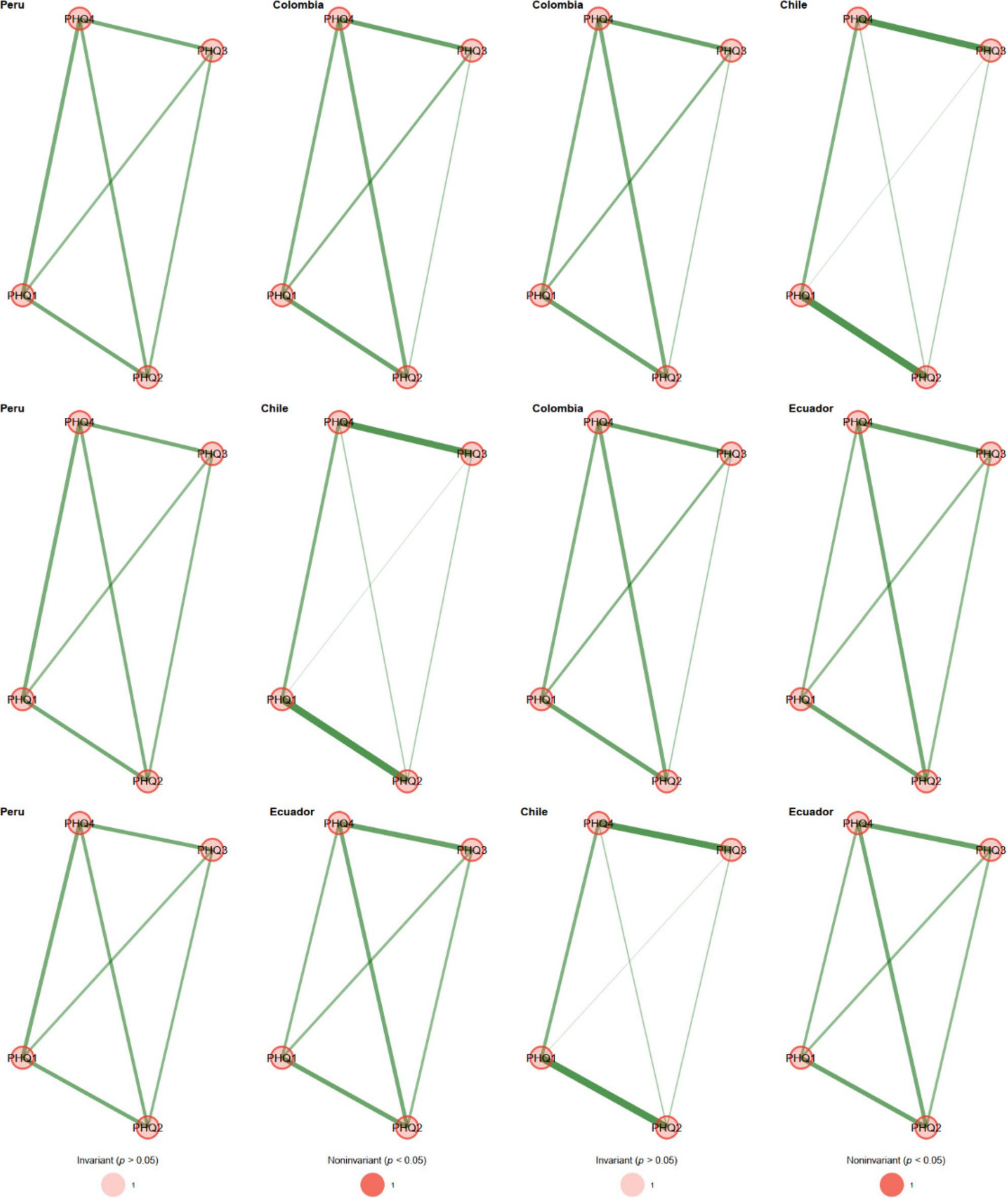
Metric invariance of the PHQ-4

Figure 3shows that in all pairwise comparisons of countries (Peru-Colombia, Peru-Chile, Ecuador-Peru, Colombia-Chile, Colombia-Ecuador, and Chile-Ecuador), the nodes were transparent, indicating compliance with metric invariance. Furthermore, Table 3 confirmed that no items showed significant differences (*p* > .05, p_BH > 0.05) in network loadings between the compared countries. These findings support the equivalent functioning of the PHQ-4 across participants from different countries, demonstrating metric network invariance.

**Table 3:** Revised network load differences by country

Items	Peru - Colombia			Peru - Chile		
	D	*p*	p_BH	D	*p*	p_BH
1	-0.03	0.51	0.84	-0.04	0.43	0.92
2	0.02	0.69	0.84	0.01	0.92	0.92
3	-0.01	0.84	0.84	0.02	0.70	0.92
4	-0.03	0.44	0.84	-0.01	0.74	0.92

Items	Ecuador - Peru			Colombia - Chile		
	D	p	p_BH	D	p	p_BH
1	0.03	0.58	0.63	-0.01	0.82	0.82
2	-0.03	0.59	0.63	-0.01	0.80	0.82
3	-0.02	0.63	0.63	0.03	0.56	0.82
4	-0.03	0.53	0.63	0.02	0.64	0.82

Items	Colombia - Ecuador			Chile – Ecuador		
	D	p	p_BH	D	p	p_BH
1	0.06	0.22	0.65	0.07	0.19	0.67
2	-0.05	0.33	0.65	-0.04	0.50	0.67
3	-0.02	0.78	0.93	-0.04	0.41	0.67
4	0.01	0.93	0.93	-0.02	0.76	0.76

Nota. D = Differences between the revised network loads; p_BH = p adjusted

## Discussion

This study provided evidence of the psychometric performance of the PHQ-4 in Peru, Chile, Ecuador, and Colombia from the perspective of Network Psychometrics. In this sense, it is one of the first studies to demonstrate evidence of cross-cultural item invariance under this new methodological approach. Network psychometrics, and networks in general, offer a methodological framework that allows for a natural and realistic study of the complexity of mental health disorders, as it allows for identifying patterns of interaction between items (symptoms) without relying on the existence of a common latent factor (Ebrahimi, 2023; Ross, 2010). In other words, the network approach posits that symptoms (items) do not measure the disorder, but rather are part of it (Cramer et al., 2010).

Following this perspective, the EGA analysis showed evidence that the items form a single community composed of four nodes in each of the four countries analyzed. Furthermore, the UVA analysis performed in each country demonstrated that each item reveals unique information about the network model. These results provide robust evidence that the four items form a single community across countries. It is important to note that the identification of a single community in network analysis does not necessarily imply that anxiety and depression represent a single latent construct, as assumed in traditional latent variable models. Rather, it reflects the pattern of associations among symptoms within the observed data.

These results are inconsistent with the findings of other studies conducted across different countries and cultures. For example, the original PHQ-4 study showed evidence of the existence of two factors (anxiety and depression) (Kroenke et al., 2009). Similarly, subsequent studies reported the existence of a two-factor model related to gender (Adzrago et al., 2024; Christodoulaki et al., 2022; Kim et al., 2021; Kocalevent et al., 2014; Kotera et al., 2024; Löwe et al., 2010; Mills et al., 2015; Obeid et al., 2024; Sanabria-Mazo et al., 2024). However, in these studies, the reported relationship between both factors was high in all countries: Greece (0.83), Germany (0.79), Saudi Arabia (0.89), Korea (0.77), the United States (0.92) and Colombia (0.83). In some countries, a two-factor structure was confirmed, but the relationship between both factors was not reported, specifically in Malaysia, Japan and Hispanics. Furthermore, most studies compared the model with a unidimensional model, which presented fit indices similar to the two-factor model, except for the RMSEA index. Regarding this index, in some studies, both models showed a poor fit to the data (Christodoulaki et al., 2022; Kim et al., 2021; Kocalevent et al., 2014). In other cases, the RMSEA index was very similar and indicated an adequate fit to the data for both the two-factor model and the unidimensional model (Löwe et al., 2010; Tan et al., 2024).

Additionally, a recent study conducted in seven European countries reported very similar results, where the relationship between both factors was high (0.87) (Kazlauskas et al., 2023). Similarly, another cross-cultural study conducted in 26 countries worldwide found a high correlation between the two factors (*r* = .88) (Apputhurai et al., 2024). About America, a study conducted in 12 Latin American countries showed that the relationship between both factors was also high in the different countries (0.77—0.87) (Caycho-Rodríguez et al. 2024a, 2024b, 2024c). Therefore, the discrepancy between the findings of the present study and those reported in previous scientific literature could be explained by the following reasons: First, the functioning of the two-factor model is very similar to the unidimensional model; therefore, some studies use or suggest using a total score of the scale to evaluate the relationship with other variables (Apputhurai et al., 2024; Kazlauskas et al., 2023; Löwe et al., 2010; Obeid et al., 2024). Furthermore, the creator of the instrument himself argues in favor of using a composite score for the entire scale (Kroenke et al., 2019). Second, the high relationship between the two factors could suggest that both dimensions may not be completely distinct and could potentially be combined into a single, more comprehensive factor. In relation to this, a recent study carried out in Paraguay using the psychometric network approach found that the four items form a single community (Caycho-Rodríguez et al. 2024a, 2024b, 2024c).

Third, the existence of a single community suggests that the PHQ-4 items are strongly related and constitute a set of dynamic symptoms that form a single system. This way of understanding the relationship between anxiety and depression is consistent with contributions made in previous literature. Several studies have shown that both constructs often coexist and have one of the highest comorbidity rates of all psychiatric diagnostic categories (Hanel et al., 2009; R. C. Kessler et al. 2005a, b; Löwe et al. 2008). Furthermore, other studies have suggested that both constructs coexist at the same point in time, in part because they share some common diagnostic criteria (Zbozinek et al., 2012). In this regard, the high comorbidity could be explained from the perspective of network theory, which postulates that there are “bridge symptoms” that can facilitate the interaction between anxiety and depression, leading to a mutual reinforcement of these disorders (Groen et al., 2020). Specifically, several studies have identified the symptoms of worry and feeling down or sad as “bridge symptoms” that link both disorders (Beard et al., 2016; Groen et al., 2020; Jin et al., 2022). Other studies have reported that anhedonia is a key symptom for the relationship between anxiety and depression (Collins et al., 2025; Winer et al., 2017). In this regard, PHQ-4 assesses the aforementioned symptoms (items 2, 3, and 4) as indicators of anxiety and depression, in addition to the symptom of nervousness. Therefore, previous studies can explain the strong relationship found between the four items and the existence of a community in the total sample and each of the countries.

These findings can also be interpreted within the broader debate between categorical approaches to psychopathology, as represented by the DSM-5, and dimensional frameworks such as HiTOP. While traditional diagnostic systems conceptualize anxiety and depression as distinct disorders, accumulating evidence suggests that these symptom domains are strongly interconnected and may reflect shared underlying processes. In this context, the HiTOP model proposes that internalizing symptoms, including anxiety and depression, are organized along a higher-order dimension that captures their common variance (Roman Kotov et al., 2017). The present findings are consistent with this perspective, as both the network analysis and the complementary assessment of practical unidimensionality indicate a strong general component underlying the PHQ-4 items. This pattern aligns with prior research demonstrating substantial overlap between anxiety and depression symptoms at both the phenotypic and structural levels (Clark & Watson, 1991; David Watson et al., 2005). From a network perspective, this overlap can be understood as the result of dense interconnections among symptoms, where activation of one symptom may increase the likelihood of others, leading to the emergence of a coherent symptom cluster (Denny Borsboom, 2017). In contrast, latent variable models interpret this pattern as the manifestation of a common underlying factor. Although these frameworks differ in their ontological assumptions, the convergence of findings across approaches in the present study suggests that anxiety and depression symptoms share a strong empirical structure, whether conceptualized as a general factor or as a tightly connected symptom network. Importantly, this convergence should not be interpreted as evidence of theoretical equivalence between models, but rather as complementary perspectives that contribute to a more comprehensive understanding of psychopathology. In this sense, the integration of network and dimensional approaches may be particularly valuable in advancing the study of transdiagnostic processes in mental health, especially in non-clinical populations where symptom boundaries tend to be less differentiated.

The present study also found that the items were stable and systematically organized into a single community 100% of the time across the four countries analyzed. These results demonstrate a high degree of stability in the identified community and guarantee the accuracy of the items in measuring anxiety and depression symptoms. Therefore, there is a high probability that the results found can be generalized to other similar samples in Ecuador, Colombia, Chile, and Peru. Furthermore, these results demonstrate a high degree of structural stability, supporting the robustness of the identified network structure. Similar results were found in a study conducted in Paraguay, which reported replication in a single community, maintaining a perfect consistency rate of 100% (Caycho-Rodríguez et al. 2024a, 2024b, 2024c).

The present study also assessed the configural and metric invariance of the instrument according to the participants’ country of origin. The results of the study showed that the PHQ-4 scale maintains a robust single-community structure; the presence and strength of edges remain similar across four different Latin American cultures. This finding demonstrates that the four groups understand the relationships between symptoms in very similar ways, allowing for valid comparisons. Ensuring the invariance of a measurement instrument across assessment groups is vital for effective intergroup comparisons. The scientific literature indicates that the EGA approach employed in this study performs comparably to existing SEM methods and, in several respects, outperforms them in detecting non-invariant items (Jamison et al., 2024). Therefore, this study contributes to a better approximation of the cross-cultural invariance of network structure at the item level.

The complementary analysis of practical unidimensionality provided additional insight into the structure of the PHQ-4. The results suggest that the instrument is dominated by a general dimension, with minimal residual multidimensionality. These findings are consistent with previous research indicating substantial overlap between anxiety and depression symptoms, and align with dimensional models of psychopathology such as HiTOP, which emphasize shared underlying processes. Importantly, this analysis should be interpreted as evidence of practical or essential unidimensionality, rather than strict unidimensionality in the latent variable sense. Furthermore, these findings are conceptually distinct from the single-community structure identified in the network analysis and are therefore interpreted as complementary rather than equivalent.

The results of this study must be interpreted considering several limitations. First, a self-report measure was used, which could increase the risk of response bias due to social desirability. It should be noted that in research and clinical practice, anxiety and depression are mostly measured with self-report instruments. However, future studies should consider obtaining data from different sources, such as relatives and friends of respondents, among others, or conducting in-depth interviews to understand anxiety and depression better. Second, the study used non-probability convenience sampling, which could limit the generalizability of the findings across the countries studied. Therefore, it is recommended that future studies employ some form of probability sampling or significantly increase the sample size in each country to ensure more reliable results. Third, the study was cross-sectional in a specific geographic area within each country. Therefore, the development of anxiety and depression over time and geographic location may be considered limitations. Future research should consider longitudinal designs and geographic areas that include more Latin American countries to clarify the findings. Fourth, an important limitation concerns the small number of items included in the PHQ-4. With only four indicators, the range of possible network configurations is inherently restricted, which may favor the identification of a single community. Therefore, the structure observed in this study should be interpreted with caution, as it may partly reflect methodological constraints rather than the true complexity of the construct. Fith, the sample consisted predominantly of non-clinical participants, with responses concentrated in the lower categories of the scale. This restricted variability may have increased inter-item correlations, potentially contributing to the emergence of a fully connected network and a single community structure.

Despite these limitations, the results of the present study provide solid evidence of the PHQ-4’s functioning in different Latin American cultures. Specifically, strong evidence was found regarding the scale’s structure, where the items form a single community and each item is relevant to the network model in each of the countries studied. Therefore, a single-community structure is supported to describe the internal organization of the PHQ-4. Evidence was also provided regarding the structural consistency and stability of the network in each country, where the items are stable and systematically organized into a single community 100% of the time. Furthermore, it has been demonstrated that the PHQ-4 items are invariant across countries. Therefore, valid comparisons can be made.

## Data Availability

Upon request authors are prepared to send relevant documentation or data in order to verify the validity of the results.
